# Production of Cloned Bighorn Sheep Embryos Using ISCNT via HMC from Domestic Sheep Oocytes Treated with Resveratrol During IVM

**DOI:** 10.3390/ani15192872

**Published:** 2025-09-30

**Authors:** José Roberto Vazquez-Avendaño, Demetrio Alonso Ambríz-García, Alfredo Trejo-Córdova, José Antonio Sandoval-Zárate, Fernando Gual-Sill, Jessica Elivier Nuñez-Macias, Fahiel Casillas, María del Carmen Navarro-Maldonado

**Affiliations:** 1 Departamento de Biología de la Reproducción, División de Ciencias Biológicas y de la Salud, Universidad Autónoma Metropolitana, Unidad Iztapalapa, Mexico City 09310, Mexico; jrva@xanum.uam.mx (J.R.V.-A.); deme@xanum.uam.mx (D.A.A.-G.); atrejo109@hotmail.com (A.T.-C.); elivier12@hotmail.com (J.E.N.-M.); fahiel@xanum.uam.mx (F.C.); 2 Dirección General de Zoológicos y Conservación de la Fauna Silvestre de la Secretaria del Medio Ambiente de la Ciudad de México, Mexico City 11850, Mexico; jaszarate105@gmail.com; 3 Departamento de Reproducción, Facultad de Medicina Veterinaria y Zootencia, Universidad Nacional Autónoma de México, Mexico City 04510, Mexico; 4 Dirección General de Vida Silvestre, Subsecretaría de Biodiversidad y Restauración Ambiental, Secretaria del Medio Ambiente y Recursos Naturales, Mexico City 11320, Mexico; f.gual.sill@gmail.com

**Keywords:** nuclear transfer, interspecies, bighorn sheep, resveratrol

## Abstract

The aim of this study was to treat sheep oocytes with resveratrol during in vitro maturation (IVM) and use them as cytoplasts in ISCNT via handmade cloning (HMC), evaluating the effect of resveratrol on the in vitro development of cloned Mexican bighorn sheep embryos. Post mortem skin fibroblasts from an adult male specimen were frozen for 8 years, thawed, and reseeded for eight cell passages. For IVM, *Ovis aries* oocytes were treated with 0, 0.5, or 1.0 µM resveratrol. Matured oocytes were manually enucleated, and triplets (*O. aries* cytoplast–*Ovis canadensis mexicana* karyoplast–*O. aries* cytoplast) were formed and electrically fused. The reconstructed embryos were chemically activated and cultured for in vitro development (IVD). The IVM rate was 81.8 ± 10.4% for CG, 81.9 ± 6.7% for EG1, and 76.3 ± 7.7% for EG2, with no significant differences between groups. For IVD, EG1 showed a statistically significant increase (*p* < 0.05) in the blastocyst rate (31 ± 12.0%) and a statistically significant decrease in the fragmented embryo rate (25 ± 10.4) when compared with the other groups. It was concluded that better rates of cloned bighorn sheep blastocysts could be obtained in ISCNT via HMC when fusing *O. aries* oocytes supplemented with resveratrol during IVM with post mortem adult male *O. c. mexicana* fibroblasts that had been cryopreserved for 8 years.

## Simple Summary

The bighorn sheep is listed on the IUCN Red List of Threatened Species. The purpose of this study was to clone bighorn sheep (*O. c. mexicana*) embryos using, as cytoplasts, oocytes from domestic sheep (*O. aries*) treated with resveratrol, an antioxidant with beneficial effects on the development of in vitro-produced embryos, and, as karyoplasts, fibroblasts from a post mortem adult male bighorn sheep from the Chapultepec Zoo, which had been frozen for 8 years. The bighorn sheep embryo clones were reconstructed using handmade cloning (HMC), and their development rate was evaluated. A higher blastocyst rate and a lower fragmentation rate were observed in cloned bighorn sheep embryos produced from sheep oocytes treated with 0.5 µM resveratrol. It was concluded that resveratrol improves the blastocyst rate and reduces fragmentation in cloned post mortem bighorn sheep embryos produced using ISCNT via HMC.

## 1. Introduction

Gene banks preserve the genetic diversity of species [1] and are essential for the conservation of sperm, eggs, embryos, cells and tissues of various mammals at risk. This is the case for the bighorn sheep *Ovis canadensis*, listed on the IUCN Red List of Threatened Species, albeit in the category of “least concern” [2]. It is subject to special protection due to factors that negatively impact its viability, such as habitat disappearance and fragmentation, illegal hunting, trafficking and trade, the introduction of exotic (domestic) species, emerging diseases, and climate change [3]. The presence of domestic species has caused the bighorn sheep to move, abandoning the areas where they were traditionally found. Therefore, there is a State Plan for the Conservation, Management, and Use of the Bighorn Sheep of Baja California Sur [4]. It is important to conserve its population to promote the conservation of current biological diversity [5].

One way to achieve this goal is through the application of animal reproduction technologies, such as embryo production through in vitro fertilization (IVF) or somatic cell nuclear transfer (SCNT), embryo freezing, and embryo transfer (ET), or a combination of them for the conservation and reproductive improvement of endangered species of economic and ecological importance [6].

Somatic cell nuclear transfer allows the production of genetically identical individuals originating from a single nuclear donor. The donor cell is a somatic cell derived in culture from the animal to be cloned and is known as the “karyoplast”. This cell is fused with an activated, nucleus-free oocyte called a “cytoplast”, or with a fertilized egg from which the pronuclei have been removed, to give rise to a cloned embryo of the desired species [7]. Both the cytoplast and the karyoplast must have specific physiological characteristics for successful SCNT (oocytes must be arrested in MII; fibroblasts must be arrested in GO/G1) and the subsequent development of the reconstructed embryo and the new cloned individual.

In Interspecies Somatic Cell Nuclear Transfer (ISCNT), in which species at risk of extinction or even already extinct are cloned using their somatic cells as karyoplasts, the cells are fused with enucleated oocytes from phylogenetically homologous or heterologous (i.e., non-homologous) domestic species [8].

A well-known case of ISCNT is the Pyrenean Ibex (*Capra pyrenaica pyrenaica*). Skin fibroblasts cryopreserved for 10 years from the last surviving goat until 2000 were thawed and fused with enucleated oocytes from a domestic goat (*Capra hircus*). The cloned embryos were transferred into hybrid and purebred female Spanish Ibex (*C. p. hispanicus*), resulting in the birth of one live offspring. This sets a good precedent for obtaining offspring from endangered or extinct species through ISCNT [9].

Other authors generated a clone of a mouflon sheep (*O. orientalis musimon*) via ISCNT using granulosa cells from females found dead in the desert, fused with domestic sheep (*O. aries*) oocytes, resulting in offspring [10]. Additionally, it was possible to clone the gaur (*Bos gaurus*), an Asian buffalo, using ISCNT from its somatic cells and domestic cattle oocytes, obtaining offspring [11].

In México, we obtained the first Mexican bighorn sheep (*O. canadensis mexicana*) embryos at the blastocyst stage, which were cloned using ISCNT through handmade cloning (HMC) from ear skin fibroblasts of the species, fused with enucleated oocytes of domestic sheep (*O. aries*) [12].

One of the challenges is the reprogramming of the karyoplast, which is already differentiated and possesses the morphophysiological characteristics and genetic imprint of the cell type in question. It is through certain treatments and, upon fusion with the cytoplast, that the karyoplast must regain its totipotentiality and state of cellular undifferentiation, thus ensuring its nuclear reprogramming [13,14,15].

Animal species gene banks store the tissues or cells of organisms for conservation or preservation; this implies the application of technologies such as cryopreservation, representing another challenge owing to the damage caused to cryopreserved cells, which affects their success rates in the development of embryos.

In ovine species [16,17], we observed that supplementation with an antioxidant (resveratrol) during in vitro maturation (IVM) of oocytes favors the development of compact morulae in embryos produced via SCNT, as well as blastocysts produced via vitrified IVF, after thawing.

Resveratrol is a phytoalexin, which acts as a defense mechanism against pathogens that affect plants and their fruits [18].

Kwak et al. [19] demonstrated its positive effect on embryonic development when it was used at 0.5 to 1 µM in porcine embryos produced through IVF.

Resveratrol reduces the levels of reactive oxygen species (ROS), which are overproduced in in vitro cultures because of incubation conditions and the handling to which the embryos are subjected. One explanation is that resveratrol increases the level of reduced glutathione (GSH). Reactive oxygen species, although necessary for certain cellular processes, cause damage to the cell (cytoplasmic and nuclear membranes) when produced in excess, which results in alterations in embryonic development such as cell blockage or death [17].

In the present study, resveratrol was used for its antioxidant activity and because it also functions as a trigger for genetic signals in embryonic development. The oocyte is known to provide an epigenetic environment for the genes necessary for embryonic development, such as *NANOG*, *Oct3/4*, and *SOX2*, which maintain pluripotency [20]. This provides a good precedent to suggest that it will be able to reprogram the karyoplast nucleus with which it will fuse during SCNT.

The objective was to evaluate its effect on the IVM of domestic sheep oocytes (*O. aries*) used as cytoplasts in ISCNT on the in vitro development (IVD) rate of cloned reconstructed bighorn sheep (*O. canadensis mexicana*) embryos, using ear skin fibroblasts of a post mortem adult male of this species that had been cryopreserved for 8 years. The innovativeness of this work is that, for the first time, Mexican bighorn sheep clone embryos are produced via ISCNT through HMC after treating domestic sheep oocytes, used as cytoplasts, with antioxidants. This study achieves double the blastocyst rates of 14 and 15% reported in the literature (there are only two previous articles, which are by Vazquez et al., 2017 [21] and Hernández et al., 2020 [12]). Thus, the results provide an alternative for increasing the production of high-quality clone embryos, which can be incorporated into germplasm banks or transferred to surrogate females as part of the management and use programs in the States of the Mexican Republic in which this species lives.

## 2. Materials and Methods

The reagents were obtained from Sigma-Aldrich Chemical Co.Ltd, Saint Louis, Missouri, USA, unless otherwise indicated. Incubation conditions were 38.5 °C, 5% CO_2_, and saturated humidity.

### 2.1. In Vitro Culture of Fibroblasts from Bighorn Sheep (O. c. mexicana)

In 2016, an 8 h post mortem (death from natural causes) adult male (5 years old) Mexican bighorn sheep (*O. c. mexicana*) was sampled at the Chapultepec Zoo in Mexico City (collection permit SGPA/DGVS/07250/15 and approval from the Ethics Commission of the Biological and Health Sciences Division, Universidad Autónoma Metropolitana Iztapalapa or UAM-I). Approximately 1 cm^3^ of its ear skin was removed and transported on ice to the Assisted Animal Reproduction Laboratory at the UAM-I within 5 h. Once in the laboratory, the tissue was kept refrigerated for 24 h before processing.

Following the methodology described by Navarro-Maldonado et al. [22], the tissue was subjected to an enzymatic disaggregation process with collagenases type I and II (0.2%/0.2%, (Gibco, Waltham, MA, USA)) in phosphate-buffered saline without calcium or magnesium (DPBS, Dulbecco’s PBS, In Vitro, S.A., Ciudad de México, CDMX, México) and incubated at 38.5 °C for 2 h under constant oscillation. The cells in suspension were washed via centrifugation and seeded in Dulbecco’s Modified Eagle Medium (DMEM, In Vitro, S.A., CDMX, México), supplemented with 10% fetal bovine serum (SFB, Microlab, S.A. de C.V., CDMX, México) and 2% antibiotic–antifungal (In Vitro, S.A., CDMX, México). For 4 weeks, the cultures with a confluence of ≥90% underwent cell passages every 7 days.

The cell passage consisted of removing the culture medium and washing three times with 1 mL of DPBS (Dulbecco’s PBS, In Vitro, S.A., CDMX, México), and then adding 1 mL of trypsin (0.05%/0.05% Trypsin-Verseno, In Vitro, S.A., CDMX, México) and incubating for 5 min or until the cells were completely detached from the base of the culture dish.

Then, 1 mL of DMEM (In Vitro, S.A., CDMX, México) supplemented with 10% FBS (Microlab, S.A. de C.V., CDMX, México) and 2% antibiotic–antifungal (In Vitro, S.A., CDMX, México) was added to inactivate the action of trypsin. The medium with the cells in suspension was placed in 1.5 mL microcentrifuge tubes (Sorenson, Biociences, Inc., Salt Lake, UT, USA) and centrifuged at 500× *g* for 5 min. The supernatant was decanted and 1 mL of DMEM was added to the cell pellet, which was reseeded in Petri dishes (Nunc, Walham, MA, USA), adding 2 mL to reach 3 mL of supplemented DMEM and incubating under the described conditions. The procedure was repeated until the cell passages were completed (5th to 9th passages), reseeding half of the cell population from each passage, and the other half was cryopreserved. For cryopreservation, the cells (fibroblasts) were stored at −80 °C for 24 h and subsequently at −196 °C for 8 years in a DMSO-based freezing medium (In Vitro, S.A., CDMX, México).

After 8 years of cryopreservation and before cloning via ISCNT, the fibroblasts were thawed and reseeded. The cryotubes were removed from the LN_2_ tank and thawed at 30 °C in a water bath; immediately after this, they were centrifuged at 500× *g* for 5 min, the supernatant was discarded, and 1 mL of supplemented DMEM was added. The samples were subsequently reseeded in 2 mL of additional medium and incubated under the conditions described. The fibroblasts were synchronized in the G0/G1 stages of their cell cycle via contact inhibition, after which they were cultured until they reached confluence (7 days). They were subsequently detached with trypsin as previously described and maintained in TCM-199 with HEPES (In Vitro S.A., CDMX, México) supplemented with 20% NBCS (Biowest, Nuaillé, France) (T20) to be used as karyoplasts for ISCNT via HMC [21].

### 2.2. In Vitro Maturation of Domestic Sheep (O. aries) Oocytes

Following the methodology described by Hernández-Martínez et al. [12], with some modifications, ovaries obtained from *O. aries* at a local slaughterhouse were collected and transported to the laboratory (transfer time of 1 h) in saline solution (0.9% NaCl and 1% antibiotic–antifungal) at 30–35 °C. Cumulus–oocyte complexes (COCs) were aspirated from ovarian follicles (2–5 mm in diameter) in TCM-199 with HEPES (In Vitro, S.A., CDMX, México) supplemented with 100 IU/mL of heparin sodium salt. The recovered COCs were selected based on their morphology and number of granulosa cell layers [23].

The samples were incubated for 22 h in in vitro maturation medium (IVM) TCM-199 supplemented with cysteine [0.57 mM], D-glucose [3.05 mM], polyvinyl alcohol [PVA] [0.001 g/mL], sodium pyruvate [0.91 mM], 10% NBCS (Biowest, Nuaillé, France), 0.1 IU of FSH-LH (Pluset, Calier, Italy), gentamicin [50 µg/mL], and epidermal growth factor [EGF, 10 ng/mL]. Three treatment groups of *O. aries* COCs were formed during IVM: the control group CG (without resveratrol), experimental group 1 EG1 (0.5 µM resveratrol), and experimental group 2 EG2 (1.0 µM resveratrol).

The criteria for the main signs of IVM evaluation were the expansion of cumulus cells and the presence of the first polar body, which indicated that the oocytes were in metaphase II (MII) [21].

### 2.3. Production of O. c. mexicana Cloned Embryos Through ISCNT via HMC

Once IVM was completed, the COC with expanded cumulus cells was incubated in 500 μL of hyaluronidase (0.5 mg mL^−1^ in TCM-199 with HEPES) for 8 min, and the oocytes were gently denuded of cumulus cells using a micropipette (200 μL). Zona pellucida-free oocytes were placed in T2 medium (TCM-199 with 2% NBCS), and oocytes with a first polar body (PB) were selected under a stereomicroscope, indicating that they were MII. Oocytes with a PB were incubated in IVM medium supplemented with demecolcine (0.5 μg mL^−1^) for 1 h under the same conditions.

For the preparation of the cytoplasts, 30 µL drops corresponding to the following solutions were placed on a 60 × 15 mm Petri dish: T2, pronase (2 mg/mL in T10), T10 (TCM-199 supplemented with 10% NBCS and 0.5 µg/mL cytochalasin B), and T20 (TCM-199 supplemented with 20% NBCS). The drops were covered with mineral oil, and cytoplasts were prepared in this dish.

Once the demecolcine incubation period was complete, the oocytes were transferred to the T2 drop on the cytoplast preparation dish. Groups of 20 to 30 oocytes were then transferred to the pronase drop for 3 min or until the zona pellucida (ZP) was completely dispersed. Immediately afterward, the ZP-free oocytes were placed in the T20 drop to inactivate pronase. This procedure was repeated for all available oocytes.

Groups of six ZP-free oocytes were distributed in each T10 droplet, and handmade cloning (HMC) was performed via manual enucleation using a microblade (Agtech, Manhattan, NY, USA). For this, the portion of the oocyte cytoplasm closest to the membrane containing the genetic material (metaphase plate and first polar body) was excised with a microblade. The enucleated oocytes (cytoplasts) were collected in the T20 droplets to restore their spherical shape as a sign of their viability.

Following this, 15 µL drops of T20, phytohemagglutinin (5 mg/mL in TCM-199 with HEPES), T2, and fusion media (0.3 M D-mannitol and 1 mg/mL polyvinyl alcohol) were placed on the lid of a 33 mm Petri dish. The drops were covered with mineral oil. Enucleated *O. aries* oocytes (cytoplasts) were immersed in phytohemagglutinin for 4 s. A single *O. c. mexicana* skin fibroblast (karyoplast) was subsequently placed between two cytoplasts, forming cell triplets.

For triplet fusion, a cell electrofusion device (Instrument BLS Budapest, Hungary) connected to a fusion chamber (BTX microslide, model 450 Holliston, MA, USA) was used, which covered the cells with fusion medium. Cell triplets (*O. aries* cytoplast–*O. c. mexicana* karyoplast–*O. aries* cytoplast) were fused using a 0.2 kV/cm pulse for 9 µs.

The reconstructed cloned bighorn sheep embryos were activated through incubation in calcium ionophore A23187 (8 µg/mL) for 5 min and in 6-dimethylaminopurine (6-DMAP 2 mM) for 4 h under the described conditions. Then, the embryos were cultured in a WOW (Well of Well) system, based on the work in Vajta et al. [24] and improved by VitaVitro (Shenzhen, China), in 50 µL of BO-IVC (IVF Biosciences, Cornwall, United Kingdom) covered with mineral oil, for 7 days (168 h) and under the described conditions [12]. At the end of the culture, the development rate for each treatment was evaluated.

### 2.4. Statistical Analysis

The data are expressed as means ± SDs. An arccosine transformation was performed on the data obtained for embryonic development. Comparisons between means for both the IVM and the different IVD stages were analyzed via ANOVA with a significance level of *p* < 0.05.

## 3. Results

### 3.1. Effect of Resveratrol on the IVM Rate of O. aries oocytes

**Figure 1:**
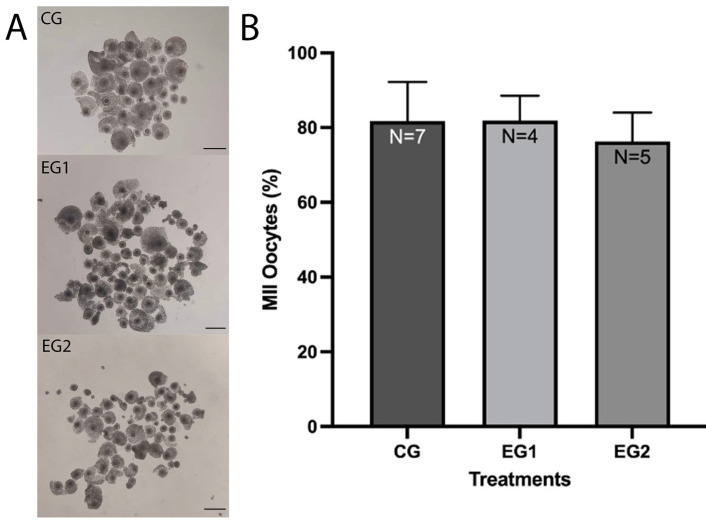
Effect of resveratrol on the IVM of sheep oocytes. (**A**) Photographs of oocytes in each treatment. Scale bar: 100 µm. (**B**) The effect of 0.0 (CG), 0.5 (EG1), and 1.0 (EG2) μM resveratrol concentrations during IVM was analyzed. Bars represent mean ± SD. N = number of replicates. Significant differences (*p* > 0.05). No letters between bars indicates no significant difference (*p* > 0.05).

A total of 357 COCs were placed in CG, 237 in EG1, and 280 in EG2. Of these, 292 in the CG (81.8 ± 10.4%), 194 in the EG1 (81.9 ± 6.7%), and 213 in the EG2 (76.3 ± 7.7%) completed IVM, with no significant differences between the groups (*p* > 0.05) (Figure 1).

### 3.2. Production of Cloned O. c. mexicana Embryos Using ISCNT via HMC

**Figure 2:**
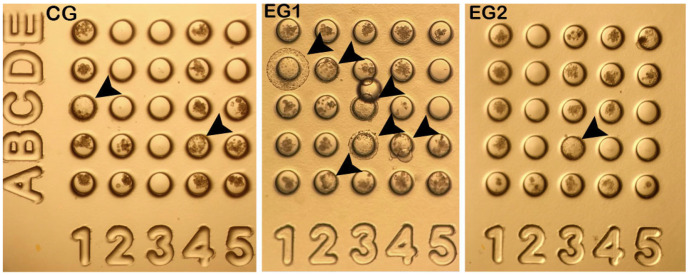
Cloned *Ovis canadensis mexicana* embryos in the WOW system on day 7 of culture, obtained from *O. aries* oocytes treated with 0.0 (CG), 0.5 (EG1), and 1.0 μM (EG2) of resveratrol during IVM. Arrowhead indicate cloned bighorn sheep embryos at different blastocyst stages: Coordinate 1C and 4B: two blastocysts in CG; 1D, 2A, 2D, 3B, 3C, and 4B: six blastocysts in EG1, three of them expanded; and 3B: one blastocyst in EG2. Magnification: 40×.

Five replicates were performed, resulting in 40, 69, and 32 cloned bighorn sheep embryos for groups CG, EG1, and EG2, respectively. Compared with CG, EG1 presented a statistically significant increase (*p* < 0.05) in the percentage of blastocysts and a statistically significant decrease in the percentage of fragmented embryos when compared with the other groups (Table 1 and Figure 2). The highest blastocyst rate was 31 ± 12.0% in EG1, followed by 16 ± 3.2% in CG and 6 ± 6.3% in EG2. The embryo fragmentation rates were 25 ± 10.4% in EG1, 42 ± 6.4% in EG2, and 46 ± 8.8% in CG.

## 4. Discussion

Although the resveratrol treatments used did not show significant differences in the IVM rates of *O. aries* oocytes, treatment with this antioxidant at 0.5 µM did have a positive effect on the blastocyst rate of cloned *O. c. mexicana* embryos produced via ISCNT. This confirms the hypothesis that the oocyte, although not homologous, provides the epigenetic environment necessary for embryonic development, since it is responsible for the reprogramming of the somatic cell by providing the transcription factors *NANOG*, *Oct3/4*, and *SOX2*, genes that maintain pluripotency [20]. In addition, the oocyte has DNA repair factors [25].

In general, embryonic genomic activation in the SCNT depends on the ability of the recipient oocyte to block transcription of the donor cell’s DNA and the corresponding translation of mRNA [8]. This allows it to reprogram the donor cell to return it to its undifferentiated, totipotent state.

To promote reprogramming of the donor somatic cell nucleus by the recipient oocyte, the cell cycles must be synchronized between the first and second stages. The somatic cell must be arrested in the G0 or G1 phase of its cell cycle, whereas the oocyte must be in MII. Arresting somatic cells in these phases involves reducing the amount of serum in the culture media; contact inhibition through cell confluence; and the use of cycloheximide, roscovitine, or dimethyl sulfoxide (DMSO) [13,14].

Another important factor is the cell passage of the donor cell, with early passages (<6–8) being preferable, since the probability of karyotype abnormalities or epigenetic alterations that may accumulate in prolonged in vitro culture is reduced [15].

In the present study, somatic cells from bighorn sheep corresponding to passages 6 to 8 were brought to confluence and exposed to DMSO during freezing and remained frozen at −196 °C for 8 years. This likely allowed them to remain in the G0 and G1 phases of their cycle, facilitating reprogramming. In addition, oocytes in the early stages of life are used, since they contain high levels of the maturation-promoting factor (MPF), which prematurely condenses chromatin, silencing the transcription of the donor cell genome after its transfer into the recipient oocyte [8].

On the other hand, resveratrol is known to act as an antioxidant by reducing the levels of ROS, a product of cellular metabolism, and increasing the level of intracellular reduced glutathione (GSH). Reactive oxygen species damage oocytes and embryos by decreasing ATP, blocking development, altering DNA methylation, and modifying histones [17].

Additionally, resveratrol is a trigger of the gene signals required for embryonic development, such as sirtuin 1 (SIRT1), a member of the sirtuin deacetylase family, which regulates the acetylation of several transcription factors and regulates cell cycle progression, in addition to promoting the cellular response to metabolic stress [26]. This SIRT1 gene participates in the regulation of mitochondrial biogenesis, ATP generation, and AMPK regulation, increasing β-oxidation and fatty acid consumption and thus improving embryonic development [27].

Resveratrol also contributes to embryonic compaction by increasing the expression of E-cadherin (uvomorulin), promoting its transcriptional activity. E-cadherin is a product of oocyte origin, a calcium-dependent molecule, and the main component of cell–cell adherens junctions. It is associated with embryonic compaction and blastocyst formation, as it plays an important role in trophectoderm differentiation by inducing polarization of the epithelial phenotype [16,28,29].

Therefore, in the present study, by adding resveratrol to the *O. aries* oocytes used as cytoplasts in ISCNT, the aim was to provide them with additional tools that would enable them to reprogram the *O. c. mexicana* cell nucleus. This possibly favored the increased rate of cloned bighorn sheep blastocysts observed when using oocytes matured in 0.5 µM resveratrol.

In previous work, resveratrol at this concentration favored the rate of compact morulae in cloned *O. aries* embryos produced via HMC [16] and blastocysts produced via IVF in this species [17].

In the present work, a decrease in the percentage of blastocysts was observed at 1.0 μM resveratrol compared to CG; however, it was not statistically significant. In studies on IVF-produced embryos from buffalo, cattle, and sheep, no differences in blastocyst rates were observed using concentrations of 0.5 to 2.0 μM resveratrol [17,30,31]. Others have observed that at high concentrations (5.0 μM), resveratrol decreases the blastocyst rate (8.1%) of sheep produced through IVF [32].

Regarding the cloning technique used, in 2013 Stroud et al. [33] cloned Rocky Mountain bighorn sheep (*Ovis canadensis canadensis*) embryos through ISCNT via traditional cloning (TC) using micromanipulators, from post mortem skin fibroblasts of an adult male fused with enucleated *O. aries* oocytes, resulting in 16% blastocysts. These results are similar to those obtained in previous studies, in which we obtained 16.1% cloned blastocysts of female *O. c. mexicana* [21] and 14.3% of male *O. c. mexicana*, using ear skin fibroblasts of live specimens as karyoplasts that were fused with HMC *O. aries* oocytes manually enucleated for HMC, but without treatment with resveratrol [12]. In the present study, by incorporating resveratrol during the IVM of *O. aries* oocytes used to clone Mexican bighorn sheep embryos, the blastocyst rate increased to 31%, surpassing previous studies. This finding is important considering that the fibroblasts also came from a post mortem specimen and from fibroblasts that had been cryopreserved for 8 years.

Loi et al. [10] reported the successful cloning of *O. orientalis musimon* via TC using *O. aries* oocytes. The authors used granulosa cells from two female mouflons found dead in the pasture. The cloned embryos reached the blastocyst stage and were transferred to *O. aries* surrogate mothers, resulting in the birth of an apparently normal mouflon clone calf.

Folch et al. [9] cloned an extinct goat, the bucardo or Pyrenean Ibex (*Capra pyrenaica pyrenaica*), from skin fibroblasts of the last specimen of the species that were fused with oocytes of a domestic goat (*Capra hircus*). The cloned embryos of the bucardo were transferred into female Spanish Ibex or hybrids (Spanish Ibex x domestic goat), obtaining offspring.

Since Vajta et al. [34] developed HMC, which involves manually enucleating the oocyte without micromanipulators, some domestic species have been cloned using this technique, such as pigs [35,36], sheep [37], cattle [38,39,40], and horses [41], as well as wild species such as buffalo [42,43,44].

Both TC and HMC have the same objective, but the instruments used to enucleate the oocytes vary. These variables include the use of micromanipulators for TC, as well as the need to use ZP-free oocytes for HMC. Furthermore, the latter requires two cytoplasts to fuse with a karyoplast, whereas the former requires only one cytoplast per karyoplast. One advantage of HMC over TC lies primarily in cost [14].

A few studies on ISCNT have been carried out in wild sheep, with cleavage rates of 84.6% for *Ovis ammon* [45] and 87.4% for *Ovis orientalis isphahanica* [46], results that are below those obtained in this study (94–100%). Regarding the blastocyst rate, most studies report rates of 7.6–15%, while Loi et al. [10] reported 30.4% in *Ovis orientalis musimon*, similarly to the data obtained in this study.

The present study reports, for the first time, the production of 31% cloned embryos at the blastocyst stage using ISCNT via HMC, from resveratrol-treated *O. aries* oocytes (as cytoplasts) and fibroblasts (as karyoplasts) that were thawed after 8 years of cryopreservation. These fibroblasts were obtained from a post mortem adult male bighorn sheep (*O. c. mexicana*), an endemic species of México that is listed on the IUCN Red List. This reinforces the feasibility of bringing extinct species back to life, as well as the ability of somatic cells to survive cryopreservation for long periods.

Although the first mammal was obtained by SCNT 29 years ago, the efficiency in obtaining live newborns remains low due to several factors, including those intrinsic to the technique itself, such as inefficient somatic cell reprogramming, limited oocyte reprogramming capacity, the intense manipulation to which the cells are subjected, and the in vitro culture conditions of the embryos [47]. In sheep, an efficiency of 5.3% to 42% cloned embryos at the blastocyst stage has been reported (revised by Vázquez-Avendaño et al., 2022 [48]). Given this situation, the implementation of antioxidant agents that enhance the quality of karyoplasts and improve the development and quality of the resulting cloned embryos is a viable option, especially if they are cloned embryos from endangered wild species. Researchers in many countries have been working on SCNT, but there are only two studies from our research group describing the production of cloned embryos in bighorn sheep [12,21].

The potential implications of the trials’ results are the innovative use of resveratrol during IVM of sheep oocytes to prepare them as cytoplasts to receive the donor cells of the species to be cloned. Resveratrol has been shown here to improve embryo development to the blastocyst stage of cloned bighorn sheep, allowing good-quality cloned embryos for the gene bank in México.

The possibility of performing ET of cloned bighorn sheep embryos in surrogate females of a homologous species in order to determine pregnancy and birth rates, as well as to evaluate the health of cloned offspring of this species, remains to be studied.

## 5. Conclusions

We concluded that supplementing *O. aries* oocytes with resveratrol during IVM, using them as cytoplasts in ISCNT, and then fusing them with post mortem adult male *O. c. mexicana* fibroblasts that had been cryopreserved for 8 years resulted in better rates of cloned bighorn sheep blastocysts.

## 6. Patents

Patent Title No. 394003 referring to Preservation of Bighorn Sheep.

## Figures and Tables

**Table 1:** In vitro development rate of cloned *O. c. mexicana* embryos produced through ISCNT using HMC from *O. aries* oocytes treated with resveratrol.

Group	No.	Cleavage N (%)	4–16 Cells N (%)	Morula N (%)	Blastocysts N (%)	Fragmented N (%)
CG	40	38 (94 ± 8.0)	6 (16 ± 18.5)	9 (21 ± 15.4)	6 (16 ± 3.2) ^a^	17 (46 ± 8.8) ^a^
EG1	69	69 (100)	12 (20 ± 22.9)	16 (24 ± 6.5)	22 (31 ± 12.0) ^b^	19 (25 ± 10.4) ^b^
EG2	32	32 (100)	8 (20 ± 19.9)	8 (26 ± 17.3)	2 (6 ± 6.3) ^a^	14 (42 ± 6.4) ^a^

Different letters (a, b) between lines indicates statistically significant differences (*p* < 0.05), while identical letters indicates no significant difference (*p* > 0.05). Five replicates were performed in each group.

## Data Availability

The data are available from the first author, José Roberto Vazquez- Avendaño (jrva@xanum.uam.mx), upon request.
